# Gene expression profiles of Protein tyrosine phosphatase non-receptor 22, Tumor necrosis factor receptor-associated factor 1 and Interleukin-1 beta in patients with rheumatoid arthritis and healthy controls after severe acute respiratory syndrome-associated coronavirus-2 infection or vaccination

**DOI:** 10.1099/acmi.0.001046.v4

**Published:** 2026-04-02

**Authors:** Maryam Hassan Sanguor, Hatham W. Atwan, Saad Waheed Mihan Alsulaitti, Iman Hussein Rabeeah, Zeenah W. Atwan

**Affiliations:** 1 Department of Nursing Techniques, Technical Institute, University of Technology, Basrah, Iraq; 2 Department of Inspection, Basrah Health Directorate, Basrah, Iraq; 3 Department of Orthopedics, Alsayab Teaching Hospital, Basrah Health Directorate, Basrah, Iraq; 4 Department of Biology, College of Science, University of Basrah, Basrah, Iraq; 5 Central Laboratory, Department of Microbiology, College of Medicine, University of Basrah, Basrah, Iraq

**Keywords:** coronavirus disease 2019 (COVID-19), IL-1β, PTPN22, rheumatoid arthritis (RA), TRAF-1, vaccination

## Abstract

**Background.** Recovery from coronavirus disease 2019 (COVID-19) probably leads to long-term symptoms, including immune system complications. Recent publications have reported that severe acute respiratory syndrome-associated coronavirus-2 (SARS-CoV-2) infection or, sometimes, vaccination against it may trigger autoimmune responses in vulnerable cohorts.

**Purpose of the research.** To investigate the differences in the expression of the Protein tyrosine phosphatase non-receptor type 22 (PTPN22), Tumor necrosis factor receptor-associated factor 1 (TRAF-1) and Interleukin-1 beta (IL-1β) genes in patients with rheumatoid arthritis (RA) and healthy controls following SARS-CoV-2 infection or vaccination.

**Methods.** Blood samples were collected from 61 patients diagnosed with RA post COVID-19 Rheumatoid arthritis patients after SARS-CoV-2 infection (RAI), and 40 controls (C) who had experienced at least one COVID-19 infection. RNA was extracted and used to prepare cDNA from each sample and was then used to analyse the expression of PTPN22, TRAF-1 and IL-1β using relative gene expression.

**Results.** The study covered all 61 patients; 17 had been vaccinated without prior COVID-19 infection Rheumatoid arthritis patients after SARS-CoV-2 vaccination (RAV), while 44 were diagnosed with RA after recovery from COVID-19 (RAI). A statistically significant decrease in the gene expression of TRAF-1 was observed in both RAI and (RAV) patients compared with the C group (*P*=0.042). A consistently significant increase in gene expression of IL-1β was observed in both RAI and RAV samples compared with controls. However, the reduction in PTPN22 expression was not statistically significant.

**Conclusion.** In this study, TRAF-1 was significantly downregulated, but IL-1β was upregulated in patients with RA post either COVID-19 infection or vaccination, while PTPN22 showed a non-significant reduction. The study findings suggest that triggered immune response post SARS-CoV-2 exposure, through infection or vaccination, may influence molecular pathways involved in RA pathogenesis.

**Impact Statement**

This study provides insight into how severe acute respiratory syndrome-associated coronavirus-2 infection or vaccination might alter the expression of key immune-related genes, thereby contributing to rheumatoid arthritis onset. By highlighting changes in IL-1β and TRAF-1 expression, the work adds valuable data to ongoing investigations of coronavirus disease 2019’s long-term autoimmune effects.

## Data Summary

All supporting data (relative expression and fold-change results for PTPN22, TRAF-1 and IL-1β) have been uploaded as a supplementary file.

## Introduction

The severe acute respiratory syndrome-associated coronavirus-2 (SARS-CoV-2) causes coronavirus disease 2019 (COVID-19). COVID-19 may be a trigger for autoimmune reactions in a subset of patients by causing significant inflammation and immune system dysfunction. Many publications have documented the presence of autoantibodies in the sera of patients with COVID-19 and autoimmune disorders following SARS-CoV-2 infection [1,3]. Arthritic symptoms following SARS-CoV-2 infection have been documented [4]. More severe symptoms were reported in patients with rheumatoid arthritis (RA), coinciding with a high increase in post-COVID-19 infection [5].

During COVID-19 infection, Cluster of differentiation 8 positive T cells (CD8+ T cells) and mature natural killer cells are overactivated, while B cells are dysregulated, and T cells and inflammatory cytokines [6] could be contributors in developing autoimmune reactions. PTPN22 and TRAF-1 are implicated in signalling that regulates T cells’ activity in the immune system. In addition, Interleukin-1 (IL-1) acts as a mediator of the inflammatory response and exacerbates tissue damage and injury during diseases.

Before the COVID-19 pandemic, several studies analysed the expression profiles of immune-related genes in patients with RA. Interestingly, reduced expression of *PTPN22* has been associated with the risk allele in individuals, which contributes to T-cell activation and autoimmune-driven inflammatory responses [7]. Consistently, decreased *TRAF-1* expression has been associated with increased production of pro-inflammatory cytokines, exacerbating joint inflammation and damage as a consequence [8]. Furthermore, *IL-1β* is considered a critical inflammatory mediator in RA pathogenesis, with elevated levels correlating strongly with synovial hyperplasia and bone erosion [9,10]. These foundational pre-pandemic insights provide an essential reference point for interpreting the post-COVID-19 immune alterations observed in the present study.

More than 200 COVID-19 vaccines were developed; several of them are under clinical trials [11]. Some of these vaccines are genetic vaccines, such as mRNA vaccines (Moderna and Pfizer/BioNTech), inactivated vaccines (Sinovac, Sinopharm, Bharat Biotech Covaxin) and viral vector vaccines (Oxford/AstraZeneca, Sputnik V). Annually, vaccinations save millions of lives and increase life expectancy. Although vaccines have been quite successful in the past 200 years, there are still some side effects, such as latent and autoimmune response through several pathways [12]. The severity and mortality of COVID-19 have decreased due to widespread vaccination. However, vaccine-related side effects, including autoimmune and autoinflammatory illnesses, such as thrombotic thrombocytopenia [13], Guillain–Barré syndrome [14], systemic lupus erythematosus (SLE) [14] and RA, have been continuously reported [15].

Therefore, the aim of this study was to investigate the role of SARS-CoV-2 infection in developing RA. The study also aimed to assess the gene expression of PTPN22, TRAF-1 and IL-1β, which are implicated in RA development.

## Methods

### Sample collection

Blood samples were collected from 61 individuals over a period of 3 months. The samples comprised Rheumatoid arthritis patients after SARS-CoV-2 infection (RAI) at the Biological Pharmaceutical Unit at Al-Basrah Teaching Hospital. The control (C) group comprised 40 individuals who were infected with SARS-CoV-2 but did not develop RA.

### Ethical approval

The study was approved by the Al-Razi Research and Development Unit, Basrah Health Directorate, Basrah, Iraq (Approval No. 462, dated 14 November 2023). All procedures were conducted in accordance with the Declaration of Helsinki, and written informed consent was obtained from all participants.

### RNA extraction and cDNA synthesis

Total RNA was extracted from whole blood using Solarbio (Cat. No. R1200, China) following the manufacturer’s recommendations. First-strand cDNA was prepared using the Universal RT-PCR Kit [Moloney murine leukemia virus reverse transcriptase (M-MLV), free Thermal equilibrium (Teq) polymerase] from Salorbio (Cat. No. RP1105, China) according to the manufacturer’s protocol and was used as a template for the downstream applications.

### Real-time PCR for genes

The specific primers for PTPN22 (forward: 5′-GATGGAGCAAGACTCAGACAC-3′; reverse: 5′-CCCCATGTTAGAAGAGCAGAT-3′) [16], TRAF-1 (forward: 5′-GCCCTTCCGGAACAAGGTC-3′; reverse: 5′-CGTCAATGGCGTGCTCAC-3′) [17], IL-1β (forward: 5′-GCACGATGCACCTGTACGAT-3′; reverse: 5′-CACCAAGCTTTTTTGCTGTGAGT-3′) [1] and Glyceraldehyde-3-phosphate dehydrogenase (GAPDH) (forward: 5′-CTTTTGCAGACCACAGTCCATG-3′; reverse: 5′-TTTTCTAGACGGCAGGTCAGG-3′) were used [18]. The real-time PCR was prepared using a kit from SolGent Co., Ltd (Cat. No. 34014, Korea). The PCR mixture comprised SYBR Green master mix (10 µl), forward and reverse primers (100 pmol), nuclease-free water (5 µl) and cDNA template (7 ng µl^−1^). The reaction protocol involved an initial denaturation at 95 °C for 10 min, followed by 35 amplification cycles, each consisting of denaturation at 95 °C for 10 s, annealing at 60 °C for 30 s and extension at 72 °C for 30 s. A final extension step was carried out at 72 °C for 5 min.

### Statistical analysis

The data were statistically analysed using a t-test (two-sample, unequal variance) at *P*≤0.05.

## Results

### PTPN22 gene expression

**Fig. 1.**
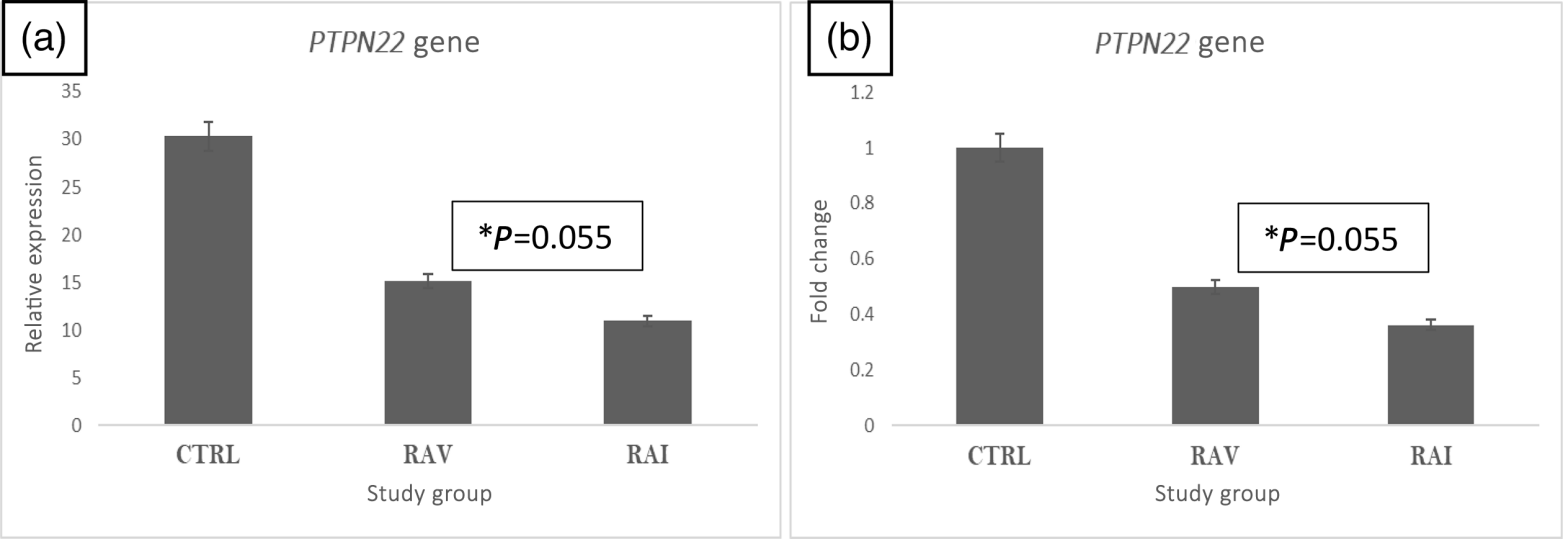
*PTPN22* relative expression (**a**) and fold change (**b**) in RAV and RAI. Following the extraction and reverse transcription of total RNA, the synthesized DNA was utilized as a template for the SYBR Green master mix Quantitative Polymerase Chain Reaction (qPCR) relative expression experiment. Data were standardized to the housekeeping gene (GAPDH) after being evaluated using Comparative threshold cycle method (ΔΔCTs) (t-test *P*=0.055, *P*=0.05 not significant). * the result is not statistically significant.

In contrast to the C samples, the fluorescence signal decreased in RAI and Rheumatoid arthritis patients after SARS-CoV-2 vaccination (RAV) samples (Fig. 1a). Normalizing the control samples to 1 showed that PTPN22 expression was reduced to 65% in the RAI group compared with the C group. Interestingly, vaccination enhanced the expression to ~50% compared with the controls (Fig. 1b).

### TRAF-1 gene

**Fig. 2.**
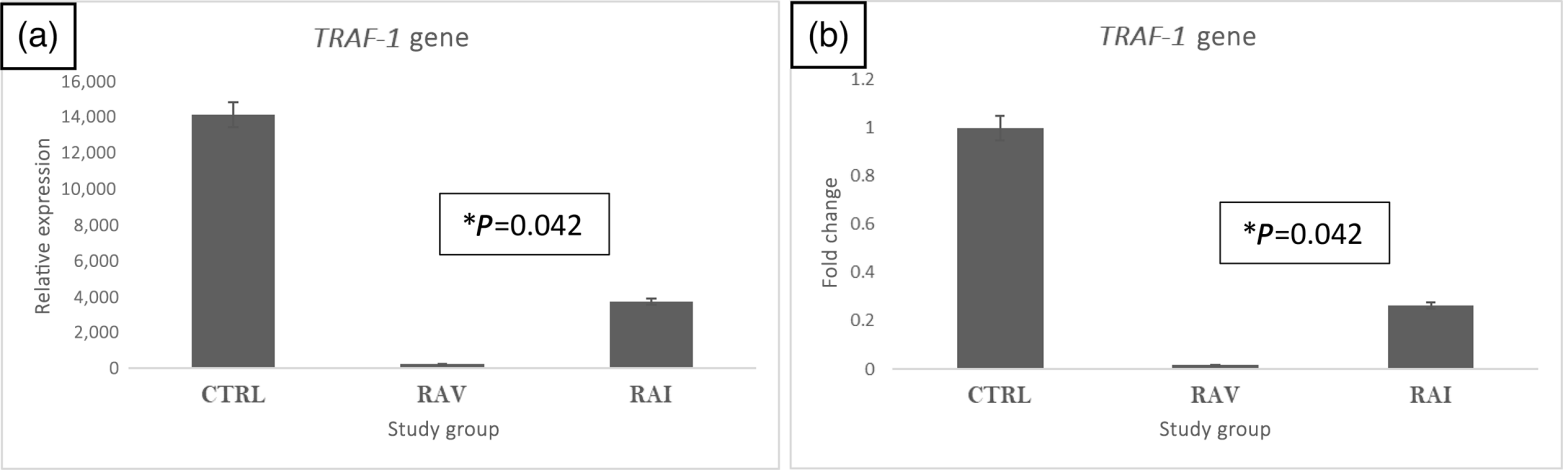
*TRAF-1* relative expression (a) and fold change (**b**) in RAV and RAI. Following the extraction and reverse transcription of total RNA, the synthesized DNA was utilized as a template for the SYBER Green master mix qPCR relative expression experiment. Data were standardized to the housekeeping gene (*GAPDH*) after being evaluated using ΔΔCTs (t-test *P*=0.042, *P*<0.05*).

TRAF-1-specific primers and SYBR Green were used to relatively assess the gene expression of TRAF-1, which is involved in many autoimmune disorders. The results showed that, after subtracting the housekeeping gene signal, TRAF-1 expression was reduced to 75% in the RAI group compared with the C group. Consistently, the expression was further reduced to almost 100% in the RAV group (Fig. 2a, b).

### IL-1β gene

**Fig. 3.**
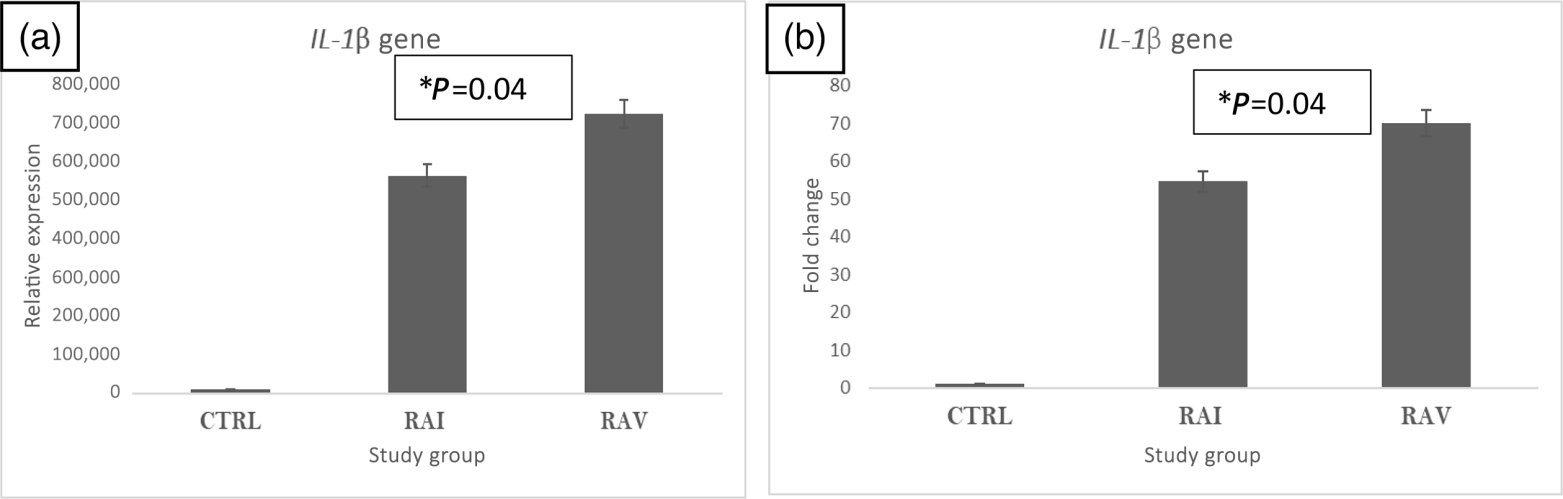
IL-1β relative expression (**a**) and fold change (**b**) in RAV and RAI. Following the extraction and reverse transcription of total RNA, the synthesized DNA was utilized as a template for the SYBR Green master mix qPCR relative expression experiment. Data were standardized to the housekeeping gene (GAPDH) after being evaluated using ΔΔCTs (t-test *P*=0.04, *P<0.05).

To quantify gene expression, prepared cDNA was used as a template in SYBR Green master mix. In contrast to control samples, it was evident that the positive fluorescence signal had increased in RAI and RAV samples (Fig. 3a). After normalizing the control samples to 1, the data were evaluated to determine the fold difference in expression, which increased in RAI and RAV to 70- and 54-fold, respectively, compared with the C group (Fig. 3b).

## Discussion

In general, viral infections are known to be associated with the development of arthritis and RA, which is diagnosed shortly after exposure to viruses [19]. Some viruses have the capacity to enhance the proliferation of human synovial cells and induce gene activation in synoviocytes [20], especially within synovial fibroblasts, which leads to the activation of pro-oncogenic genes and genes encoding pro-inflammatory cytokines. These genes include IL-1, IL-6 and TNF-α, which eventually result in synovial hyperplasia and inflammation [20].

To evaluate the possible involvement of PTPN22 in RA development after SARS-CoV-2 infection, we examined its gene expression profile and observed a reduction in RAI samples compared with controls. However, this decrease did not reach statistical significance (*P*=0.055), suggesting a need for a larger number of patients, which probably proves the significant difference. This observation is still important, since many previous studies have shown similar downregulation of PTPN22 in autoimmune diseases such as SLE [7,21], indicating that subtle changes in its expression may contribute to immune dysregulation.

Decreased PTPN22 gene expression in patients with RA carrying the risk allele (rs2488457 and rs2476601) leads to an inflammatory response, since T-cell activation is not repressed by this gene, and decreased PTPN22 augments the development and symptoms of RA in a mouse model [22]. Decreased or absent PTPN22 expression leads to an adverse immune response and increased disease severity in patients with SLE [23]. One suggested mechanism could be the persistent activation of antigen-specific, class II-restricted Cluster of differentiation 4 positive T cells (CD4+ T cells) along with other cell types triggered by PTPN22 deficiency, which eventually leads to the development of inflammation in the synovium.

PTPN22 is also implicated in the uptake and presentation of autoantigens mediated by vimentin–dectin-1 interaction and cytokine release. Citrullinated vimentin-specific serum autoantibodies in patients with RA stimulate osteoclastogenesis and bone resorption [24]. However, the results disagree with Chang *et al*. [25], who reported that patients with SLE expressed higher levels of PTPN22 than controls [25]. Another indication implicating PTPN22 in arthritis is that PTPN22 interacts with neutrophil cytosolic factor 1 (Ncf1) and is modulated by oxidation through the noncatalytic C129 residue; oxidation-prone PTPN22 contributes to the increased severity of T-cell-dependent autoimmunity development [26]. Similarly, PTPN22 gene expression was decreased in RAV samples compared with C. Vaccination did not make any difference in terms of the gene expression of the PTPN22, which could be interpreted as having no role in changing the inflammatory response of PTPN22.

Given the critical role of TRAF in cytokine production, the relative expression of TRAF-1 was analysed, showing a significant decrease in the RAI group compared with the control group. The results are consistent with Mirzaesmaeili and Abdul-Sater [25], who found that injecting mice with TRAF-1-deficient macrophages instead of WT macrophages resulted in worsened tissue damage, immune cell infiltration and joint inflammation. In fact, TRAF-1 limits the linear ubiquitination of the adaptor apoptosis-associated speck-like protein apoptosis-associated speck-like protein containing a CARD (ASC), which reduces IL-1β release and prevents the nucleotide-binding oligomerization domain (NOD)-like receptor protein 3 inflammasome from assembling in macrophages [27].

In general, the expression of TRAF-1 in patients with RA is low, which leads to a significant and increased production of pro-inflammatory cytokines [3,8]. Additionally, decreased expression of TRAF-1 within cells is associated with fatigue of CD8+ T cells, leading to a higher viral load during the chronic stage of viral infection. Initially, TRAF-1 expression is high in CD8+ T cells, and as the viral infection progresses, the level of TRAF-1 expression decreases. This is attributed to a decrease in the ability of CD8+ T cells to eliminate virus-infected CD4 cells [3].

However, the results do not align with the conclusions of Qiao *et al*. [28] and Cheng *et al*. [8], who found that TRAF-1 expression was elevated in inflammatory bowel tissue and synovial tissue of patients with RA [5,28].

IL-1β is classified as one of the components involved in autoimmune inflammatory disorders, including RA [9], and this was the reason it was included in this study. The results showed a significant increase in IL-1β expression in RAI samples. The results are consistent with Cañete *et al*. [15], who found an increase in interleukin-1 beta in patients with RA. Excessive elevation of IL-1β also contributes to the severity of the disease in immune diseases [29]. Production of IL-1β due to infection of epithelial cells with SARS-CoV-2 acts as a pro-inflammatory cytokine and is released following inflammasome activation [30,31].

In fact, IL-1β is an effective inducer of bone resorption through receptor activator of NFκB ligand receptor activator of nuclear factor kappa-B ligand (RANKL) overexpression, which promotes osteoclastogenesis. IL-1β upregulates the NFκB-mediated extracellular signal-regulated kinase / signal transducer and activator of transcription 1 (ERK/STAT1) signalling pathway, which, in turn, augments the expression of inflammatory factors [16,32]. Kay and Calabrese [33] found that excessive IL-1β in the knees of rabbits leads to joint damage with histological features of RA. Vaccination slightly reduced the expression profile of IL-1β to ~23%. That suggests an important role for vaccination in correcting the expression of RA-implicated genes.

## Conclusion

The study demonstrated downregulation of PTPN22 and TRAF-1 gene expression in patients with RA following SARS-CoV-2 exposure, suggesting potential changes in immune response pathways rather than a reduced biological role. In contrast, IL-1β expression was significantly elevated, emphasizing its key contribution to inflammation and bone resorption. These findings indicate that IL-1β may serve as a possible molecular link between SARS-CoV-2 exposure and the pathological processes of RA.

## Supplementary material

10.1099/acmi.0.001046.v4 Uncited Table S1.

## Abbreviations

COVID-19: coronavirus disease 2019
qPCR: quantitative polymerase chain reaction
RA: rheumatoid arthritis
RAI: rheumatoid arthritis patients after SARS-CoV-2 infection
RAV: rheumatoid arthritis patients after SARS-CoV-2 vaccination
SARS-CoV-2: severe acute respiratory syndrome-associated coronavirus-2
SLE: systemic lupus erythematosus
ΔΔCT: comparative threshold cycle method
